# A Case of Isolated SARS-CoV-2 Fulminant Myopericarditis Without Respiratory Failure

**DOI:** 10.7759/cureus.14003

**Published:** 2021-03-19

**Authors:** Felix Afriyie, Emmanuel Fohle, Sammir S Dekowski, Shruthi Kumar

**Affiliations:** 1 Internal Medicine, East Carolina University/Vidant Medical Center, Greenville, USA; 2 Internal Medicine, University of North Dakota, Fargo, USA; 3 Internal Medicine, St. George’s University School of Medicine, St. George’s, GRD

**Keywords:** sars-cov-2 and covid-19, cardiogenic shock, myopericarditis, acute myopericarditis, fulminant myopericarditis

## Abstract

Coronavirus disease 2019 (COVID-19) is an infectious disease caused by severe acute respiratory syndrome coronavirus 2 (SARS-CoV-2). Several cardiovascular complications of COVID-19 have been described in clinical studies. While those with pre-existing cardiovascular disease seem to have worse outcomes, growing evidence suggests that COVID-19 itself can cause myocardial injury, arrhythmia, and heart failure. We report a case of a 27-year-old male with no known comorbidities who presented with nausea, vomiting and non-radiating substernal chest pressure without respiratory symptoms in May of 2020. Laboratory findings showed elevated cardiac biomarkers and electrocardiogram showed diffuse ST-segment elevation. Coronary angiography revealed normal coronaries but findings suggestive of cardiogenic shock. Reverse transcription polymerase chain reaction for SARS-CoV-2 returned positive. He was treated for fulminant myopericarditis and cardiogenic shock with remdesivir, steroid, inotropes and vasopressors but rapidly deteriorated and went into cardiac arrest and was unable to be resuscitated despite multiple rounds of cardiopulmonary resuscitation. Fulminant myopericarditis is a rare complication of COVID-19 with high mortality that requires early recognition, treatment and a transfer to a tertiary facility with advanced cardiac services.

## Introduction

Coronavirus disease 2019 (COVID-19) is an infectious disease caused by severe acute respiratory syndrome coronavirus 2 (SARS-CoV-2). The first case of COVID-19 was reported in Wuhan, China, in December 2019 and it was declared a global pandemic on March 11, 2020, by the World Health Organization (WHO) [1]. Despite the fact that respiratory failure is the principle form of severe COVID-19, cardiac involvement is common including heart failure, myocardial infarction, arrhythmia, stress-induced cardiomyopathy, coronary vasospasm, and myocarditis. Fulminant myopericarditis (FM) is a rare condition characterized by sudden and extreme cardiac inflammation, which often results in death due to cardiogenic shock (CS), ventricular arrhythmias, or myocardial infarction [2,3]. We present a case of a reverse transcription polymerase chain reaction (RT-PCR)-confirmed COVID-19 in a young patient without pre-existing cardiovascular disease who developed FM that rapidly progressed to CS and cardiac arrest.

## Case presentation

**Figure 1:**
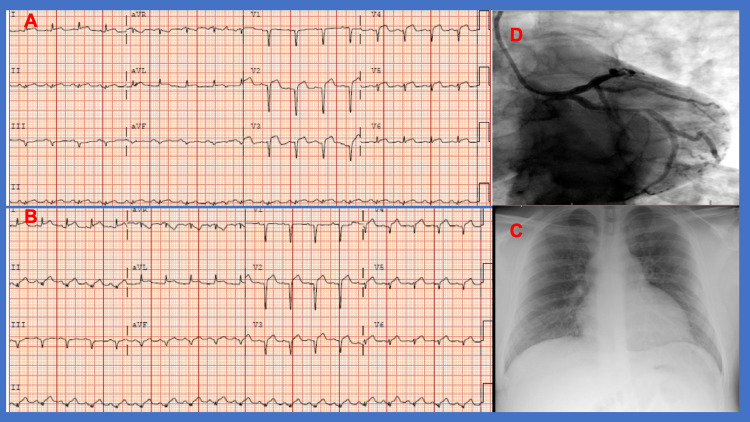
(A) Diffuse ST-segment elevation; (B) EKG one hour later showed no changes in ST elevation; (C) chest x-ray with mild pulmonary vascular congestion; (D) cardiac angiography with patent coronary vessels

A 27-year-old male with no known cardiovascular disease was transferred to our hospital for a higher level of care. He first presented to an outside hospital emergency department in May of 2020 with symptoms of nausea, vomiting, and sudden non-radiating substernal chest pressure. At the outside facility, his vital signs included a heart rate of 86 bpm, blood pressure of 103/86 mmHg, temperature of 36.6 °C (97.8 °F) and pulse oximetry of 100% SaO_2_. Initial laboratory tests showed mild leukocytosis, negative urine toxicology, and elevated troponin I and B-type natriuretic peptide (BNP). The electrocardiogram (EKG) showed diffuse ST-segment elevation without reciprocal ST changes (Figure 1A). He was quickly transferred to our facility for further evaluation and management. Upon arrival in our facility, the patient rapidly decompensated and became hypotensive. Repeat laboratory tests are summarized in Table 1. Physical examination was notable for diaphoresis with cold extremities and edema, and tachycardia with mild elevated jugular venous pressure (JVD). Respiratory examination revealed clear breath sounds bilaterally. Repeat EKG one hour later showed no changes in ST elevation (Figure 1B). Chest x-ray revealed mild pulmonary vascular congestion but no consolidation (Figure 1C). The patient underwent emergent coronary angiography that revealed normal coronaries, severe biventricular heart failure and CS with cardiac output of 2.1 L/min (normal range 4.0-8.0 L/min), cardiac index of 0.9 L/min/m^2^ (normal range 2.5-4.0 L/min/m^2^), pulmonary artery pulsatility index of 0.5 (normal >2.5), cardiac power output of 0.4 W, and pulmonary artery saturation of 46% (normal range 70%-75%) (Figure 1D).

 

**Table 1:** Repeat laboratory findings at the time of admission RBC, red blood cell; WBC, white blood cell; BUN, blood urea nitrogen; ALP, alkaline phosphatase; ALT, alanine transaminase; AST, aspartate aminotransferase; BNP, B-type natriuretic peptide; FEU, fibrinogen equivalent units

	Admission	Reference range
Hemoglobin	19	13-15 g/dL
RBC	3.39	4.6-6.8 x 10^6^/mcL
WBC	11.02	3.6-10.3 x 10^3^/mcL
Platelet	207	140-420 x 10^3^/mcL
Blood glucose	220	70-100 mg/dL
Sodium	137	135-145 mmol/L
Potassium	4.3	3.7-5.1 mmol/L
Chloride	100	96-110 mmol/L
Bicarbonate	20	22-32 mmol/L
BUN	33	6-24 mg/dL
Creatinine	1.93	0.6-1.3 mg/dL
Calcium	8.1	8.5-10.5 mg/dL
Bilirubin total	0.2	0.2-1.2 mg/dL
ALP	156	30-150 U/L
ALT	76	0-35 U/L
AST	40	0-35 U/L
D-dimer	1,215	220-500 ng/mL FEU
Troponin I	11.52	0.0-0.028 ng/mL
BNP	667	0-100 pg/mL

His clinical presentation was suggestive of FM with cardiogenic shock. Sputum was collected and examined for 18 viral nucleic acids related to the respiratory tract, but all returned negative. The RT-PCR assay of the nasopharyngeal swab returned positive for SARS-CoV-2. Echocardiogram revealed ejection fraction of 15% with severely depressed right ventricular systolic function. The patient was started on remdesivir, methylprednisolone, inotropic support with dobutamine and milrinone drip, but his condition continued to decline requiring intubation, pressor support with norepinephrine, epinephrine and admission to the intensive care unit. Circulatory assistance devices with peripheral venous-arterial extracorporeal membrane oxygenation (va-ECMO), percutaneous left ventricular assist device (LVAD) or intra-aortic balloon pump (IABP) were considered along with a transfer to an advanced facility, but unfortunately the patient became progressively hypotensive and went into pulseless electrical activity (PEA) arrest and was unable to be resuscitated despite multiple rounds of cardiopulmonary resuscitation (CPR).

## Discussion

SARS-CoV-2 is an enveloped, positive-sense single-stranded ribonucleic acid (sRNA) that enters the human host cell by binding to angiotensin-converting enzyme 2 (ACE2) [4,5]. The ACE2 receptors are membrane proteins that can be found on ciliated columnar epithelial cells of the respiratory tract, type II pneumocytes, and cardiomyocytes [5]. A cytokine storm affecting multiple organ systems, direct myocardial cell injury through ACE2 receptors, and myocardial oxygen supply and demand mismatch are all possible mechanisms for cardiac involvement [6]. Acute myocardial infarction, acute heart failure, CS, myocarditis, and arrhythmia are among the cardiac manifestations that have been reported [4]. FM is a rare condition mainly caused by a variety of viral infections and characterized by sudden diffuse cardiac inflammation and rapid occurrence of CS with a need for hemodynamic support [2]. Only few cases have been reported since the start of the pandemic but often with concomitant respiratory failure.

Clinical presentations of COVID-19-induced myocarditis or myopericarditis vary widely. It can manifest itself in different ways, from mild symptoms such as fatigue, dyspnea including chest pain and moderate ventricular dysfunction to life-threatening arrhythmia and serious heart failure requiring mechanical circulatory support [7]. Diagnostic evaluation includes elevated cardiac biomarkers, images such as echocardiogram or cardiac magnetic resonance (CMR) but endomyocardial biopsy (EMB) remains the gold standard [8]. EKG tracings may show sinus tachycardia, left bundle branch block, decreased QRS wave amplitude, ventricular premature beat, ST elevation, all of which are signs of extreme progression [9]. Echocardiography results can vary according to the state of the FM patient’s heart function [10]. Troponin I or T, and also BNP or N-terminal pro-BNP (NT-proBNP) are myocardial injury markers and can be elevated in both acute coronary syndrome and FM [7]. Coronary angiography is used to distinguish these and it is important to differentiate between them as the treatment regimens are completely different [7,11]. The key parts in the management of patients with FM include immunosuppressive therapy with glucocorticoid and supportive measures with circulatory assist devices such as ECMO, LVAD, or IABP to reduce wall stress and inflammation [2,12,13].

In our case, we observed an increase in cardiac biomarkers for myocardial injury with ST-segment elevation on EKG and marked systolic dysfunction in the echocardiogram and patent coronary vessels. CMR was not done due to acute illness and neither was EMB due to lack of consent during coronary angiography. Due to a lack of clinical trials that provide good scientific evidence in cases of COVID-19-related fulminant myopericarditis, and the uncertainty of COVID-19 treatment with hydroxychloroquine and azithromycin in the early stages of the pandemic, these drugs were not used, but the patient received steroid in addition to remdesevir, inotropes, and pressure support. While the use of circulatory assist device was being considered, along with a potential transfer to a high-level center, the patient went into PEA arrest and was unable to be resuscitated despite multiple rounds of CPR.

## Conclusions

Even though respiratory failure is the cardinal form of severe COVID-19 infection, this case highlights the importance of understanding the lethal cardiac complication of COVID-19 infection, its presentation, diagnosis, and treatment. Myocardial injury can present with variable clinical manifestation. Fulminant myopericarditis is a rare life-threatening condition that requires immediate attention. During this pandemic, frontline health care providers’ education and awareness of this condition is crucial to increase timely access to adequately resourced facilities, including a transfer to a tertiary center with specialized cardiac team if necessary, to avoid multiorgan failure and to tailor specific therapy as early as possible in the disease process.
